# A Pan-European Review of Good Practices in Early Intervention Safeguarding Practice with Children, Young People and Families: Evidence Gathering to Inform a Multi-disciplinary Training Programme (the ERICA Project) in Preventing Child Abuse and Neglect in Seven European Countries

**DOI:** 10.1007/s42448-022-00132-x

**Published:** 2022-11-15

**Authors:** J. V. Appleton, S. Bekaert, J. Hucker, G. Zlatkute, E. Paavilainen, H. Schecke, M. Specka, N. Scherbaum, E. Jouet, L. Zabłocka-Żytka, M. Woźniak-Prus, J. Cz. Czabała, S. Kluczyńska, B. Bachi, F. Bartoli, G. Carrà, R. M. Cioni, C. Crocamo, H. E. Rantanen, M. Kaunonen, I. Nieminen, L. Roe, K. Keenan, G. Viganò, A. Baldacchino

**Affiliations:** 1 Oxford, UK; 2 Oxford School of Nursing and Midwifery, Faculty of Health and Life Sciences, Oxford Brookes University, Oxford, UK; 3 School of Medicine, University of St Andrews, St Andrews, UK; 4 Faculty of Social Sciences/Health Sciences Unit, Etelä-Pohjanmaa Hospital District, Tampere University, Tampere, Finland; 5 LVR-Hospital Essen, Department of Addictive Behaviour and Addiction Medicine, Medical Faculty, University of Duisburg-Essen, Essen, Germany; 6 Laboratoire de Recherche en Santé Mentale, Et Sciences Humaines Et Sociales, Groupe Hospitalier Universitaire Paris Psychiatrie & Neurosciences (GHU- PARIS), Paris, France; 7 Department of Psychology, The Maria Grzegorzewska University, Warsaw, Poland; 8 Department of Psychology, University of Warsaw, Warsaw, Poland; 9 Department of Medicine and Surgery, University of Milano-Bicocca, Milan, Italy; 10 School of Geography and Sustainable Development, University of St Andrews, St Andrews, UK; 11 Synergia S.R.L. and Department of Decision Sciences, Università L. Bocconi, Milan, Italy; 12 Formerly Oxford Institute of Nursing, Midwifery and Allied Health Research (OxINMAHR), Faculty of Health and Life Sciences, Oxford Brookes University, Oxford, UK; 13 Faculty of Social Sciences/Health Sciences Unit, Pirkanmaa Hospital District, Tampere University, Tampere, Finland

**Keywords:** Child maltreatment, Safeguarding, Child protection, Multidisciplinary, Training, European

## Abstract

Child maltreatment has detrimental social and health effects for individuals, families and communities. The ERICA project is a pan-European training programme that equips non-specialist threshold practitioners with knowledge and skills to prevent and detect child maltreatment. This paper describes and presents the findings of a rapid review of good practice examples across seven participating countries including local services, programmes and risk assessment tools used in the detection and prevention of child maltreatment in the family. Learning was applied to the development of the generic training project. A template for mapping the good practice examples was collaboratively developed by the seven participating partner countries. A descriptive data analysis was undertaken organised by an a priori analysis framework. Examples were organised into three areas: programmes tackling child abuse and neglect, local practices in assessment and referral, risk assessment tools. Key findings were identified using a thematic approach. Seventy-two good practice examples were identified and categorised according to area, subcategory and number. A typology was developed as follows: legislative frameworks, child health promotion programmes, national guidance on child maltreatment, local practice guidance, risk assessment tools, local support services, early intervention programmes, telephone or internet-based support services, COVID-19 related good practices. Improved integration of guidance into practice and professional training in child development were highlighted as overarching needs. The impact of COVID-19 on safeguarding issues was apparent. The ERICA training programme formally responded to the learning identified in this international good practice review.

## Introduction

All children have the right to be free from all forms of violence (UNICEF, 1989). Child maltreatment can lead to enduring negative physical and mental health consequences for individuals and families, and detrimental outcomes for a country’s economic and social development. World-wide, nearly 3 in 4 children aged 2–4 years suffer physical and psychological maltreatment from family and carers (WHO, 2020). Child maltreatment has been identified as a global public health issue. According to the World Health Organization’s most recent European Report on Preventing Child Maltreatment, there is a prevalence of 9.6% for sexual abuse, 22.9% for physical abuse and 21.9% for mental abuse (Sethi et al. 2013). Psychological and social consequences for individuals include impaired physical and mental health, relationship difficulties, and poorer school and work performance (Abbasi et al., 2015). Child maltreatment also impacts a country’s economic and social development, health and social care, education, crime, youth justice costs, and worker productivity (Conti et al., 2017).

The World Health Organization has developed a policy framework for the European region to support knowledge exchange on good practice to prevent child maltreatment (Sethi et al. 2013). The ERICA project, *Stopping Child Maltreatment through Pan-European Multiprofessional Training*, responds to the call for prevention and international collaboration. The ERICA project sought to develop an international training programme to increase the knowledge and skills of non-specialist threshold professionals to prevent, detect and respond to suspected or identified child maltreatment. ‘This was a three-step process: training needs identification, the development of the training modules and materials, piloting and evaluating the training programme, resulting in the finalised ERICA training programme (Zlatkute et al., 2021, p. 4).’ This unique project has been coordinated by teams in seven European countries (Finland/Principal Investigator, England, France, Germany, Italy, Poland and Scotland). It was funded by the Rights, Equality and Citizenship Programme of the European Commission (European Commission 2019–2021).

This paper reports on learning from a rapid review of good practice examples in the prevention of child maltreatment across the seven participating countries. As defined by the WHO in the European report on preventing child maltreatment (WHO, 2013), good practices are cost-effective prevention programmes, based on strong evidence, shared experience and intersectoral action. This was one of several preliminary stages of the ERICA project to inform the training programme aims, content and delivery. A systematic literature review exploring family members’ perspectives on relationships with social care was also undertaken and is reported separately (Bekaert et al., 2021). A full protocol for the ERICA training programme is available (Zlatkute et al., 2021, psyarxiv.com/7qe5c).

## Aims and Objectives

The aim of the review was to identify core aspects of good practice examples in the detection and prevention of child maltreatment in the family across the seven participating countries. The process of identifying, mapping and learning from good practices described here ensured programme development drew on a broad international evidence base that includes examination of practice alongside peer reviewed literature and could be replicated in programme development generally. Good practice review ensures learning from real world examples. These examples were drawn from team members’ expert knowledge and other leading professionals and networks in each country. The focus was to gather a range, rather than an exhaustive pool, of data to explore common elements of good practice, to which the ERICA training project could respond. Examples were sought that: related to multi-professional working practices in preventing child maltreatment, supported practitioners’ knowledge and skills on how to identify risk of child maltreatment, and included risk assessment tools in child protection work. The team established a pan-European evidence base of programmes tackling child maltreatment, including those that involved different agencies and professional groups, and engaged the community in practical strategies to prevent and intervene effectively where child maltreatment is present. The review included purposefully seeking out programmes targeting low threshold and non-specialised services which were less likely to have specific child protection training for their role.

## Methodology/Method

A template for mapping the good practice examples was developed through discussion and expert consensus with the ERICA team in each of the seven countries, and drew on some team member’s expertise and involvement in the Camille project^1^ (Tabak et al., 2016; Viganò et al., 2017). The team included doctors, nurses, public health nurses, psychologists and educationalists. The team were asked to primarily focus on programmes tackling child maltreatment in the broadest sense, including strategies to engage the local community in the protection of children, preventive programmes and the promotion of quality child-parent relationships. As this work involved the analysis of existing materials available in the public domain, ethics committee approval was not required.

Whilst cognisant of the WHO (2013) definition of good practice, we utilised a broader definition in this review and included practices defined by child maltreatment experts, family organisations and legal/government/administrative authorities, as well as promising practices/interventions. All country partners were requested to consult with local experts about programmes and tools in existence and with user or family organisations. ‘Experts’ included experts in child health, e.g. paediatricians, researchers, educationalists, mental health practitioners. Each country was also asked to include statutory good practices, i.e. guidance, training or actions to take that are put in place through legislation and policy, such as compulsory training programmes required by government authorities and mandatory reporting. This was in addition to non-statutory examples such as regional programmes and national support systems. All examples were analysed. The aim was to achieve an overview, with a range of examples in the areas cited above, and draw out evidence to inform the ERICA training development.

Good practice examples were gathered, examined and organised into three overarching domains. These domains corresponded to those implemented at a national, local and practitioner level:

1. *Programmes tackling child abuse and neglect* in the broadest sense, including strategies to engage the local community in the protection of children, preventive strategies, and the promotion of quality child-parent relationships. For example, universal interventions such as home visiting or positive parenting programmes.
2. *Local practices and interventions in how to assess and refer if a child is at risk of abuse and/or neglect.* These included working methods used to evaluate the child’s situation such as what to do when risk is identified or known. This might involve the use of national or local guidance and multi-professional ways of working.
3. *Risk assessment tools* used by practitioners or specific services.

The mapping template focussed on specific aspects from identified good practice: a description and context including the setting; population group and professional groups involved; factors around implementation; positive outcomes; funding source; strengths/limitations; evidence base; evaluation. These areas could then be descriptively analysed across the sections, i.e. what were the target groups represented, mostly specialist professionals or lay people; and gaps identified, i.e. training for non-specialist practitioners. Key findings were identified using a thematic approach.

## Findings

**Fig. 1:**
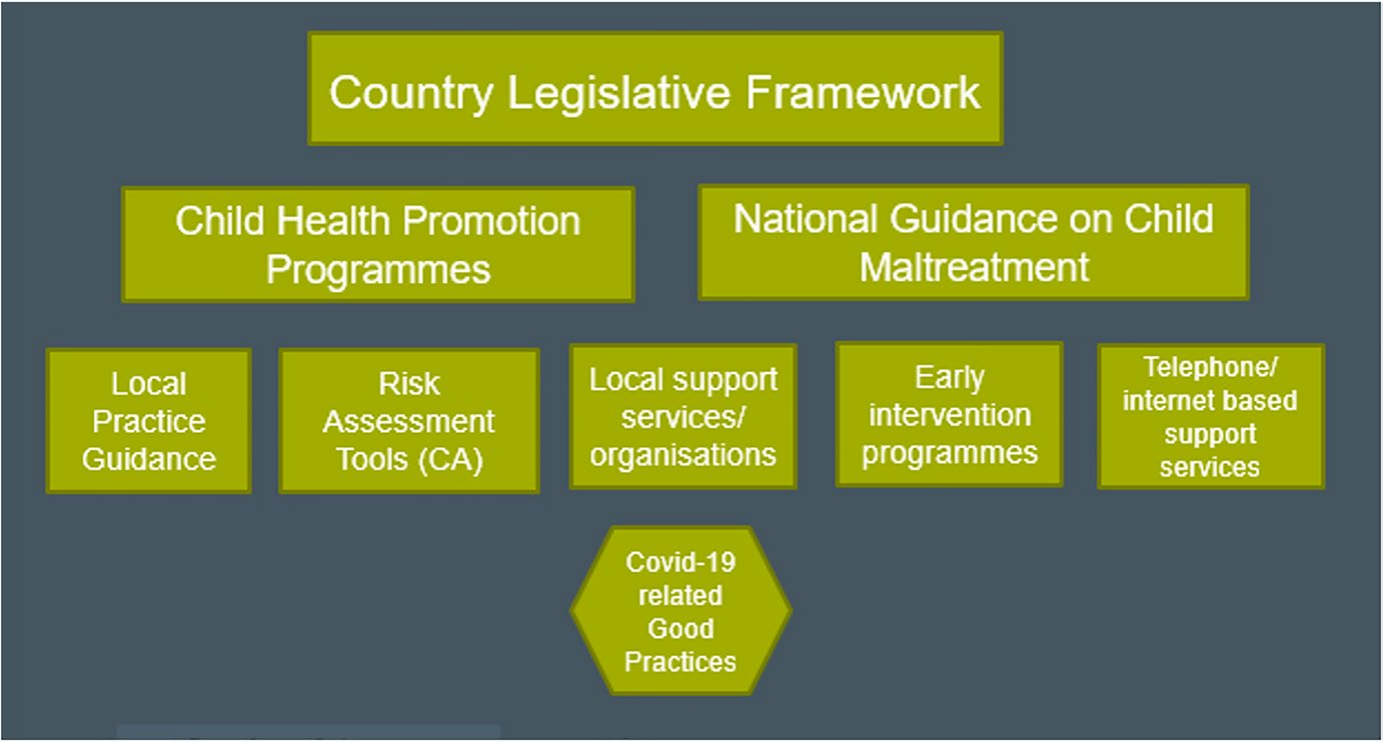
Typology identified from the good practice examples

A total of 72 good practice examples were gathered across the European teams, organised into the three domains: national programmes tackling child abuse and neglect, local practices and interventions, risk assessment tools. Further subcategories were developed within each domain, with one standalone research study in its own domain (Table 1). Through reflective discussion, a typology was developed (Fig. 1) which included: country legislative frameworks, child health promotion programmes, national guidance on child maltreatment, local practice guidance, risk assessment tools, local support services and organisations, early intervention programmes, telephone- or internet-based support services, and COVID-19-related good practices.

**Table 1:** Pan-European categorisation of good practice examples according to domains, subcategory and number

Good practices	72
Programmes tackling child abuse and neglect	45
National Child Welfare/Children Acts (2)/national guidance on child maltreatment and identification (11)	13
Early intervention/preventive programmes in families, schools and communities	13
National Child Health Promotion Programme	8
National phone hotlines for parents, professionals and children in danger (5) and government body analysing calls (1)	6
Organisation awarding national standards of child protection to education and care institutions	1
National programme to help children stay safe from sexual abuse	1
COVID developments — Triple P programme (parenting support) (1), national parenting support hotline (1), webportal of resources (1)	3
Local practices and interventions	21
Local public Social Work services (3) and Child Protection prevention committee (1)	4
Organisations providing support to families/parents in crisis/stress (2), for children in vulnerable situations (1) runaway Young People (YP) (1)	4
Local guidance/protocol on what to do if child abuse suspected	3
Organisations providing specialist services for child sexual abuse	3
Trauma recovery programme (1), project on witnesses of domestic violence (DV) (1), DV support (1)	3
Child welfare organisation/mother and baby home (1), family centres (1)	2
Peer support network for YP leaving care	1
Family therapy for parents	1
Risk assessment tools	5
Risk assessment tool	3
Structured assessment model	2
*(Research study)*	*1*

The majority of examples (45) were categorised to the areas of legislation and national guidance, including child health promotion programmes. National frameworks for child protection exist in all partner countries to guide child protection practice. For example, *The Children Act *1989 and^ 2004^ are the key legislation for England, and in Scotland, the *Children (Scotland) Act *1995*.* A range of good practices were reported which focused on country-specific child health promotion programmes. These were *universal early intervention and public health programmes*, available to all children and their families. These included Germany’s Early Childhood Intervention (ECI) programme (*Frühe Hilfen*) (Renner et al., 2018) and the Finnish national guidelines for maternity and child health care clinics, providing free services for all children and their families, produced by the National Institute for Health and Welfare of Finland. Such programmes mandate important opportunities for practitioners working with children and families to undertake child health needs assessments. This includes children who may be at risk of child maltreatment or violence in the home.

*National guidance* existed in all countries to inform professionals working with children, young people and their families to safeguard and promote child welfare and to identify child maltreatment. For example, in England, *Working Together to Safeguard Children* (HM Government, 2018) and in Scotland, *Getting It Right for Every Child* (The Scottish Government, 2017) are the statutory guidance. Finland has the *National Guidance on Child Maltreatment Identification* (Paavilainen & Flinck, 2013); (an update focusing on risk of child maltreatment is planned for 2022). Finland has also adopted the Barnahus model that is a child-friendly, multidisciplinary and interagency model for responding to child violence and witnesses of violence. In Germany, there is a national evidence-based guideline for child protection addressing maltreatment, sexual abuse and neglect among children (Deutsche Gesellschaft für Kinderschutz in der Medizin 2019 in Wißmann et al., 2019). In Poland, a national ‘Blue Card’ procedure to coordinate assistance for families experiencing domestic violence (Grzyb, 2020). In Italy, CISMAI (Coordinamento Italiano Servizi Contro Il Maltrattamento E L’Abuso All’Infanzia/the Italian Network of Agencies against Child Abuse) is an association that acts as a reference point for all the protocols and guidelines in Italy on child safeguarding and protection, for example guidance regarding conflictual parental separation (CISMAI, 2019).

There were twenty-one examples of *local practice guidance and locally based support services* to prevent child abuse and neglect reported across all countries. For example, the City of Paris in France has funded specialised ‘prevention clubs’ with workers providing support to young people aged 12 to 21. In Senigallia, Italy, a project called ‘Famiglia Forte - L’unione fa la forza’/Good Practices for the Protection of Minors in schools helps identify children in school who might be at risk of abuse at home. This network brings together representatives from local health, social care, education and law enforcement organisations to discuss difficult cases where there are concerns about a child or young person’s safety and wellbeing. The *Local System for the Prevention of Child Abuse in Warsaw*, Poland, is a programme of interdisciplinary cooperation implemented at the city level, whose main goal is the prevention of child abuse. Some countries identified locally developed guidance for child protection practice that responded to national guidance. For example, in Germany, alongside the nationwide early intervention services for families with children from 0 to 3 years of age, each of the 16 federal states also has their own child protection policies.

Five *risk assessment tools* were identified to assist practitioners. For example, Germany has a risk assessment tool developed by the National Centre of Early Intervention which provides a structured template for health care professionals (Systematische Explorations- und Verlaufsinventar für Gesundheitsfachkräfte in den Frühen Hilfen/Systematic Exploration and Process Inventory for health professionals in early childhood intervention services (SEVG)) (Scharmanski & Renner, 2016). Northern Italy has a training manual: the first professional’s manual ‘Il 1° quaderno del professionista’ (Brambilla & Masi, 2014). The manual provides medical and non-medical staff with specific guidance on the detection of different forms of child maltreatment and provides information on the procedures staff should undertake when maltreatment is suspected or identified. In England, the Framework for the Assessment of Children in Need and their Families offers guidelines for a holistic approach to assessments for families where children may be in need of protection or support (DoH, 2000).

Some countries offer *telephone and internet-based support systems* for situations where a child is in danger or at risk of abuse or neglect. These are for professionals, members of the public, and children and young people. For example, in France, 119 is the National Call Service for Children in Danger, and in the UK, a national telephone hotline, Childline, exists for children and young people up to their 19th birthday which is provided by the NSPCC (National Society for the Prevention of Cruelty to Children) charity. In Germany, there is a National Child Protection hotline specifically for all health professionals who suspect child maltreatment (Heimann et al, 2021).

During the analytic process, a specific category was identified relating to good practices implemented as a *result of COVID-19*. In Italy, the CISMAI website developed a portal dedicated to information and resources relating to child maltreatment arising from the social, economic and emotional impact of COVID-19. In France, a free national parenting support hotline (France 22) was launched in response to COVID-19. The Triple P - Positive Parenting Programme (Sanders, 2008) which runs in 25 countries across the globe, developed guidelines to support the flexible delivery of their evidence-based parenting programmes. Alternative delivery modes such as online or telephone were suggested in response to practitioners’ and family members’ movements being restricted during the pandemic.

## Discussion

A wide range of good practices were reported across the seven participating countries. Legislation was present in each country. There were national universal policy and guidelines, alongside projects that responded to local need. There was variation in the population group focus of the local practice/intervention, risk assessment tool or programme - child, young person, family, child and family or practitioner. Some programmes had several elements embedded within projects: responding to the varied needs of children, families and communities. Most examples in all three domains (programmes tackling child abuse and neglect, local practices and interventions in how to assess and refer if a child is at risk of abuse and/or neglect, risk assessment tools) tended to be geared towards registered practitioners such as nurses, doctors or psychologists.

Discursive analysis highlighted areas for development at a national, local, and practitioner level. For example, some local interventions/projects were time limited, i.e. not available at weekends or reduced hours, which can leave children and families vulnerable if a need arises ‘out of hours’. Further funding could increase consistent availability of support. It was noted that outcome data was not consistently gathered, particularly at a local level. If systems were in place to gather this data, an evidence base to support provision would be created. It was noted that some professional guidance could be long, detailed and tended not to be ‘user friendly’. A need for guidance to be more clearly related to, and better integrated into, practice and training was identified. A need for training in child development was also identified to enable practitioners to understand expected developmental stages, and when this might be hindered or interrupted through maltreatment or neglect. Resource gaps were identified for specific individuals within the family unit: young people in particular. Whilst legislation and guiding frameworks were universally present, few risk assessment tools to assist professionals working directly with families were highlighted in the review. The data gathering exercise coincided with the COVID-19 pandemic. The pandemic caused major disruptions to the lives of children and their families across the world (Appleton & Sidebotham, 2020), and the impact was consistently present in team discussions as the safeguarding issues arising from serial lockdowns became apparent.

## Recommendations

**Fig. 2:**
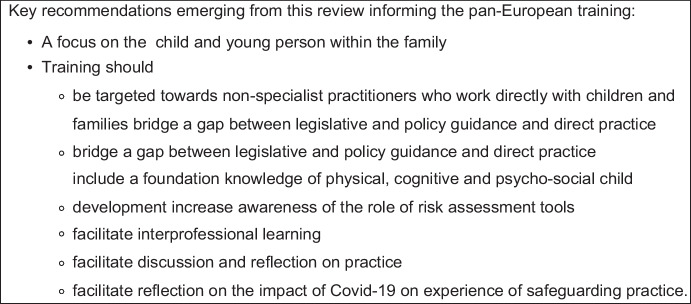
Summary of learning from the good practice review informing the ERICA training programme

The aim of this pan-European good practice review was to seek examples in prevention of child abuse and neglect in the family across the seven participating countries, and apply the learning to development of the ERICA training project. Whilst some findings were relevant to macro-systems such as universal provision and funding, the ERICA training project’s role was to respond to practitioner-facing gaps and recommendations. As a result of the review and wider baseline work, the training was targeted towards non-specialist practitioners working directly with children and families. The training project content would focus on bridging the gap between legislative and policy guidance, and direct practice with children and families. As a response to the findings of this good practice review, content would include a foundation knowledge of typical physical, cognitive and psycho-social child development to inform maltreatment risk assessment. Risk assessment tools would be discussed and their role in aiding dialogue with families, and assessment and referral to specialist support where indicated. The training would also facilitate interprofessional learning, discussion and reflection on practice relating to the core training areas. The training was originally planned to be delivered in-person; however, it soon became apparent with the impact of COVID-19 restrictions that this would not be possible and an online interactive modality was developed. Evaluation of this aspect of the training showed this approach to be effective. In a separate paper, the digital approach to the ERICA training was shown to be an acceptable, interactive and alternative approach to training regarding child maltreatment during critical times such as a pandemic (Crocamo et al, 2022). There would also be reflective space within the training to explore and discuss the impact of COVID-19 on participating practitioners’ experience of child maltreatment prevention practice. Figure 2 outlines a summary of learning from the good practice review informing the ERICA training programme.

The ERICA training was developed, piloted and revised across 2020–2021. The programme was launched in November 2021. It is available as a generic EU training package and as a country specific training package (specific to the seven participating countries) via the following weblink: (https://www.entermentalhealth.net/ericatraining).

## Footnotes

1 A pan-European training package for professionals in how to support children who have parents with a mental illness or struggle with substance misuse.
